# Non-Celiac Gluten Sensitivity and Protective Role of Dietary Polyphenols

**DOI:** 10.3390/nu14132679

**Published:** 2022-06-28

**Authors:** Nadia Calabriso, Egeria Scoditti, Marika Massaro, Michele Maffia, Marcello Chieppa, Barbara Laddomada, Maria Annunziata Carluccio

**Affiliations:** 1 Institute of Clinical Physiology (IFC), National Research Council (CNR), 73100 Lecce, Italy; nadia.calabriso@ifc.cnr.it (N.C.); egeria.scoditti@ifc.cnr.it (E.S.); marika.massaro@ifc.cnr.it (M.M.); 2 Department of Biological and Environmental Sciences and Technologies (DISTEBA), University of Salento, 73100 Lecce, Italy; michele.maffia@unisalento.it (M.M.); marcello.chieppa@unisalento.it (M.C.); 3 Institute of Sciences of Food Production (ISPA), National Research Council (CNR), 73100 Lecce, Italy

**Keywords:** polyphenols, immunity, intestinal barrier, gut microbiota, inflammation, gluten, NCGS, wheat sensitivity, bound polyphenols, bioactive compounds

## Abstract

Pathogenetically characterized by the absence of celiac disease and wheat allergy, non-celiac gluten sensitivity (NCGS) is a clinical entity triggered by the consumption of gluten-containing foods that relieved by a gluten-free diet. Since it is very difficult to maintain a complete gluten-free diet, there is a high interest in discovering alternative strategies aimed at reducing gluten concentration or mitigating its toxic effects. Plant-based dietary models are usually rich in bioactive compounds, such as polyphenols, recognized to prevent, delay, or even reverse chronic diseases, including intestinal disorders. However, research on the role of polyphenols in mitigating the toxicity of gluten-containing foods is currently limited. We address the metabolic fate of dietary polyphenols, both as free and bound macromolecule-linked forms, with particular reference to the gastrointestinal compartment, where the concentration of polyphenols can reach high levels. We analyze the potential targets of polyphenols including the gluten peptide bioavailability, the dysfunction of the intestinal epithelial barrier, intestinal immune response, oxidative stress and inflammation, and dysbiosis. Overall, this review provides an updated overview of the effects of polyphenols as possible dietary strategies to counteract the toxic effects of gluten, potentially resulting in the improved quality of life of patients with gluten-related disorders.

## 1. Introduction

Wheat is one of the most important cereals, providing energy and nutrients to billions of people around the world. In addition to its nutritional value and ability to grow in a wide range of environmental conditions, another reason for wheat’s success relies upon gluten, which has unique properties, allowing the dough to give rise to a variety of products such as bread, numerous baked products, and pasta. However, wheat gluten is also responsible for inducing a number of intolerances. Over the past two decades, there has been a steady growth in the incidence of diseases believed to be associated with gluten/wheat ingestion [1]. This trend is likely due to a significant improvement in diagnostic tools, but an actual increase in disease incidence has also been registered [2]. The reasons for this rise are not yet fully understood. Some issues could be responsible, such as the occurrence of microbial dysbiosis in the gut, changes in dietary habits, or the use of gluten as an ingredient in the preparation and processing of different food types [3,4,5]. In general, the spectrum of gluten-related disorders is wide, including celiac disease, wheat allergy, and non-celiac gluten sensitivity (NCGS). Celiac disease is an enteropathy with autoimmune features triggered by gluten-containing foods in susceptible individuals [2]. Wheat allergy is characterized by the production of IgE antibodies against wheat proteins and the development of symptoms of immediate-type food allergy [6]. Among gluten-related pathologies, NCGS has been “rediscovered” in recent years because of its growing incidence. NCGS is a condition characterized by intestinal and extra-intestinal symptoms related to the ingestion of gluten-containing foods, and it is relieved by a gluten-free diet (GFD) in patients in whom celiac disease and wheat allergy have been excluded [1,7,8]. Gluten-related diseases are now a serious public health problem that can be overcome by adherence to a GFD. Because it is very difficult to maintain a complete GFD for extended periods, there is great interest in exploring alternative strategies aimed at reducing gluten concentrations in foods or mitigating the toxic effects of gluten on humans.

Polyphenols are the most common plant-derived bioactive components in our diet. They are present in a variety of foods such as fruits, vegetables, cereals, and legumes and beverages such as tea, coffee, and wine, and they are recognized to exhibit health-promoting properties. These compounds have been demonstrated to exert health benefits thanks to their antioxidant activity and also antibacterial, iron-chelating, anti-inflammatory, and immunomodulatory properties [9,10]. According to their chemical structure, polyphenols are subdivided into several classes including phenolic acids, stilbenes, lignans, flavonoids, and other polyphenols [11]. There is evidence that polyphenols are bioactive dietary agents able to prevent, delay, or even reverse chronic inflammation while supporting tolerance and tissue repair in a variety of inflammatory diseases, including intestinal disorders [12]. However, research on the role of polyphenols in mitigating the toxicity of gluten-containing foods is currently limited.

The purpose of this review is to provide an up-to-date overview of the role of wheat components in triggering gluten/wheat-related syndromes, including the difference in gluten content and composition between ancient and modern wheat varieties. In addition, the potential protective mechanisms of polyphenols in reducing gluten bioavailability and mitigating gluten-related symptoms will be addressed, including the effect of polyphenols on innate immunity, inflammation, intestinal barrier function, and dysbiosis.

## 2. Variation for Gluten Content and Composition between Old and Modern Wheat Varieties

There is a widespread belief that wheat breeding programs have affected gluten content and composition, resulting in a higher incidence of celiac disease, wheat allergies, and NCGS. For these reasons, the scientific community compared modern and old wheat varieties from different growing areas in the world.

### 2.1. Wheat Proteins

Based on their function, wheat grain proteins are classified in structural, functional, or storage proteins, the relative abundance of which is controlled in a spatio-temporal way during grain development and grain filling [13]. Overall, protein content and composition depend on the genotype but also on a wide range of environmental factors, such as nitrogen fertilization, rainfall, and irrigation during grain filling [14].

Among the structural/functional proteins, albumins accumulate mainly in the embryo and aleurone cells, being deputed to the control of a number of metabolic processes including starch and proteins accumulation in the grains [15]. Albumins and the storage globulins, also occurring in the embryo and aleurone compartments, represent only 15% of total wheat proteins but are the ones with the highest content of essential amino acids. Other functional amylase/trypsin inhibitors (ATIs) protect the grains from insects and fungi. Accounting for 2–4% of the total wheat grain proteins, the ATIs can be responsible for non-celiac wheat/gluten sensitivity inducing intestinal inflammation [16]. The great majority of wheat proteins, over 80% of the total, are the storage proteins, namely the gliadins and the glutenins [15], which contribute to form the gluten, a proteinaceous network conferring unique viscoelastic properties to the bread and durum wheat doughs [17].

### 2.2. Variation in Protein Content and Composition in Old and Modern Wheat

A wide range of variability exists in the wheat germplasm in terms of protein content and composition as a result of the long history of wheat evolution, domestication, and breeding [18,19]. As a result of selection and breeding activities, the seed protein percentage in old and modern wheat varieties is lower compared to that of the wild wheat progenitors [20,21]. This was especially due to the selection for improving the yield components and to a negative correlation between yield and protein content [22].

**Figure 1:**
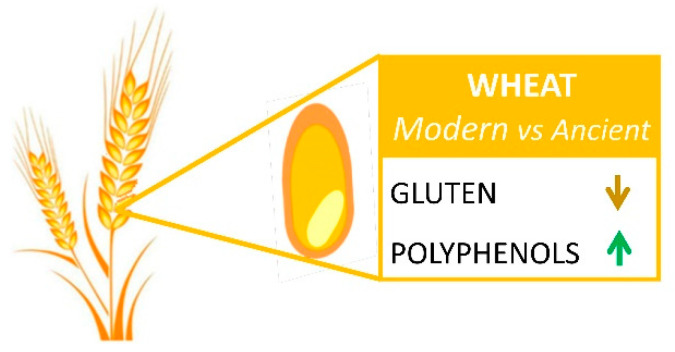
Variation trend in gluten and polyphenols content in modern and ancient wheat.

Comparative studies of old and modern wheat cultivars were also carried out to assess the variation for protein content and composition [23]. In the temporal range between 1900 and 1990, the Italian and Spanish durum wheat varieties underwent a decrease of grain protein concentration, dropping from about 18% of old varieties to an average of 14% of modern cultivars [20,24]. Analogous declines were observed in bread wheat cultivars released from 1870 to 2013 [21,25,26]. When protein composition was considered, gliadin contents also showed a decrease over time, contributing to lowering the celiac immunotoxicity of modern cultivars as compared to old varieties and wild wheat progenitors (Figure 1) [24,27,28]. In fact, the glutenin/gliadin ratio and B-type LMW-GS was improved in modern wheat genotypes [29], while the α-type and γ-type gliadins triggering celiac disease did not show significant differences between old and modern durum wheats [29]. Moreover, a significant reduction of ω-type gliadins was observed in modern cultivars, especially of ω-5 gliadin, which is an important allergen in wheat-dependent exercise-induced anaphylaxis [29]. Nevertheless, the information on ATI variations is still limited, though some preliminary studies showed a low variability [30].

### 2.3. Gluten Digestibility

Wheat gluten is resistant to complete digestion in the human gastrointestinal tract due to the high content of prolines and glutamines that characterize both the gliadins and glutenins. The digestion of these storage proteins gives rise to large peptides, some of which (i.e., 13-, 19-, and 33-mer) contain amino acid sequences, called epitopes, that cause inflammatory responses associated with celiac disease [31]. A preliminary study considered a set of 36 modern bread wheat varieties from European markets and 50 wheat landraces [32]. The materials were compared for the occurrence of two celiac disease epitopes, namely Glia-α9 and Glia-α20. The presence of the Glia-α9 epitope was higher in the modern cultivars than in the landraces; nevertheless, the results also showed that some genotypes had low contents of both the epitopes, independently if they were modern or ancient [32]. Subsequent works analyzed gluten peptides generated by in vitro digestion of old and durum and bread wheats, bread, and wild wheat accessions [33]. The authors found that old genotypes gave rise to higher contents of peptides with immunogenic and toxic sequences compared to the modern ones. More recently, the production of gluten peptides from in vitro digestion was analyzed by Ficco et al. [34] in a larger number of durum wheats. The variability for peptide profiles was larger in old genotypes than in the modern ones. In particular, some γ-gliadin peptides that trigger the adaptive immune reaction and a few α-gliadin peptides that are toxic to celiac patients were more abundant in some old varieties [34]. Similar findings were reported by Šuligoj et al. [35], revealing that all tested genotypes caused wide ranges of stimulation indices independently of ploidy level or year of release, evoking heterogeneous small intestinal T-cell responses [35]. Moreover, the authors concluded that immunogenicity should be tested considering gluten-specific T-cell lines from a large number of celiac patients to better evaluate the actual lower toxicity of wheat genotypes. Finally, recent studies showed that even though old wheat varieties give rise to fewer immunogenic peptides after human digestion ex vivo, they are still highly toxic to celiac patients [36].

### 2.4. Studies on Phenolic Acids Variation in Wheat

Phenolic acids are the major class of phytochemicals in wheat. Typically, they are grouped as hydroxy derivatives of either cinnamic or benzoic acid. The former types are the most common and among others include ferulic, sinapic, and *p*-coumaric acids; the latter group comprises a number of minor components, such as vanillic, syringic, and *p*-hydroxybenzoic acids [37]. The concentration of phenolic acids in whole grains is influenced by environmental conditions and by grain types, varieties, and the part of the grain sampled [38]. Higher concentrations of phenolic acids are found in the outer tissues of the kernel, the aleurone layer having the highest concentration, especially of ferulic and sinapic acid. Conversely, the starchy endosperm displays the lowest contents of all phenolic acids [39]. As structural components of plant cell walls, phenolic acids are involved in ester bonds with arabinose units of cell wall arabinoxylans and are the precursors of monolignols playing a key role in the formation of secondary walls.

Phenolic acids are classified also as free soluble, soluble conjugates bound to low molecular weight compounds, and bound insoluble forms linked to cell wall polymers by ester bonds. In the wheat grains, the majority of phenolic acids, corresponding to 75–80% of the total, occur as insoluble bound forms, and 20–25% are esterified to sugars and other low molecular mass compounds, whereas only 0.5–2% are soluble free [40]. Free forms are efficiently absorbed by the intestine, whereas the bound are poorly metabolized both in the stomach and small intestine [41]. Nevertheless, several studies showed that ester- and ether-linked forms are released in the intestine by gut microbiota, resulting in a higher bioavailability and biological activity [42]. Phenolic acids are scavengers of free radicals, which are primarily responsible for the oxidative damage caused to DNA, lipids, and proteins [43]. The antioxidant activity of phenolic acids depends typically on the number of hydroxyl groups on the benzene ring and ortho-substitution with the electron donor methoxy group [44]. Associated with endogenous antioxidant mechanisms, phenolic acids reduce endothelial dysfunction and inflammation [45]. Moreover, biofortified bread extracts rich of phenolic acids showed vascular health properties by downregulating the LPS-induced expression of chemokines and pro-inflammatory cytokines in endothelial cells and monocytes [46]. Phenolic acids also protect against carcinogenesis [47]. Yet, further evidence is required for this class of phytochemicals to result in health claims approved by FDA or EFSA [48]. To improve the content of phenolic acids in wheat end-products, different milling by-products were used as supplemental ingredients in pasta- and bread-making processes [49,50]. Moreover, conventional breeding was considered as an alternative option to increase the content of phenolic acids in raw materials. To this aim, the wheat germplasm was screened for the variability for phenolic acids. Results showed a common phenolic acid profile between bread and durum wheat species and about a 3.5-fold content variation across the genotypes [51]. Differences between old and modern wheat varieties were noted with respect to the qualitative/quantitative composition of some minor components (Figure 1) [40,52], and higher contents of phenolic acids were found in modern durum cultivars compared to old cultivar, wild progenitors, and landraces [51,53].

## 3. Epidemiology, Diagnostic Criteria, and Physiopathology of NCGS

NCGS, also called gluten sensitivity, gluten intolerance, or non-celiac wheat sensitivity, was first described in 1978 but did not gain recognition by clinicians until the 21st century. It is characterized by intestinal and extra-intestinal symptoms associated with the ingestion of gluten-containing foods, in subjects who are not affected by either celiac disease or wheat allergy, as per the original Salerno experts’ criteria definition [54]. The most common gastrointestinal symptoms reported by patients with NCGS are abdominal pain, bloating, and changes in bowel habits. The most common extra-intestinal symptoms include tiredness, headache, foggy mind, anxiety, musculoskeletal, and skin manifestations, which are also common in patients with disorders of gut–brain interaction [54]. Gastrointestinal symptoms are more common than extra-intestinal ones and could appear hours or days after intake of grain components [55]. The prevalence of NCGS continues to be heterogeneous, ill-defined, and based on a limited number of studies. The main limitation of NCGS prevalence data refers to the lack of reliable diagnostic biomarkers. Most studies have evaluated the prevalence of NCGS on the basis of self-reported diagnoses, which could be strongly influenced by various factors, including cultural and dietary differences. A correct estimate of the prevalence of NCGS could only be achieved by applying double-blind controlled studies, but this would be impracticable on a large scale. Taking these limitations into account and based only on self-reported data, the prevalence of NCGS is estimated to be between 0.5% and 15% [56]. It is higher in the third to fourth decade of life and generally higher in women than in men [57]. It is believed that NCGS is more widespread in individuals with a history of autoimmune and functional gastrointestinal disorders [58,59]: autoimmune diseases are indeed present in 24–25.3% of subjects with a diagnosis of NCGS [60]. However, patients do not exhibit villous atrophy or produce specific antibodies or IgE in response to ingestion of wheat. Antibodies to native gliadin have occasionally been associated with NCGS but have not shown sufficient diagnostic accuracy. In addition, NCGS appears to be associated with microscopic enteritis (in 50% of patients), neurological disorders, the presence of anti-nucleus antibodies, and first-degree relatives with celiac disease [61].

The understanding of NCGS has evolved since the Salerno experts’ criteria, and we recognized that the spectrum of symptoms may occur not just due to the ingestion of gluten proteins but also due to other potential wheat-related components. Evidence also suggests that wheat components other than gluten, such as fermentable oligo-, di-, monosaccharides, and polyols (FODMAPs) and ATIs, could also act as triggers of some clinical manifestations in NCGS cases, either intestinal or extra-intestinal or both [62]. On this basis, some authors have indicated that the terminology “non-celiac wheat sensitivity” can be more appropriate, as components other than gluten could be implicated in NCGS disease [63]. The symptoms of NCGS widely overlap with those reported by patients suffering from irritable bowel syndrome and functional dyspepsia. Several potential predisposing factors have been documented, but the molecular mechanisms or genetic bases that link NCGS with other disorders are not clear to date.

### 3.1. Current Knowledge on NCGS Pathogenesis

The pathogenetic mechanisms responsible for NCGS are not fully understood but are thought to be very complex and to reveal a multifactorial origin, with the innate immune response playing a predominant role.

The overall idea is that gliadin can cause a transient increase of the intestinal permeability irrespective of disease status [64]. Increased intestinal permeability favors the access of gliadin and other macromolecular antigens into the lamina propria, where the host responds with first-line defense mechanisms such as neutrophil recruitment to the site of exposure [65]. Several studies have reported altered expression of innate immune components in response to gluten/wheat consumption in wheat-sensitive individuals, including mucosal Toll-like receptor-2 (TLR-2) [66], peripheral blood mononuclear cells-derived interleukin-10 (IL-10), granulocyte colony stimulating factor (GCSF), transforming growth factor-α (TGF-α), and chemokine CXCL-10 [67]. A reduced expression of forkhead box P3 (FOXP3), which is a marker of T-regulatory cells, has also been found in intestinal biopsies from NCGS patients in comparison to healthy subjects and celiac patients [58,68]. An elevated production of interferon-γ (IFN-γ) has been shown in NCGS patients upon gluten challenge [69]. Besides the involvement of the innate immune system, several data also support a role of the adaptive immune system in NCGS, as suggested by an increase of anti-gliadin antibodies (AGA) in approximately 50% of NCGS patients [70], though the lack of specific markers, such as anti-TG2 antibodies and anti-deamidated gliadin antibodies, rather points to a mechanism that is different from that in celiac disease. Several studies have also reported intestinal inflammation occurring in NCGS patients, including increased levels of eosinophils, intraepithelial CD3+ T cells, and lamina propria CD45+ cells [71] and a slight increase of intraepithelial lymphocytes, although NCGS patients do not show an altered villous architecture as seen in celiac disease cases [71]. Recent evidence also suggests an intestinal barrier dysfunction in NCGS patients, although some data are conflicting. In a first study, compared to celiac and dyspeptic control subjects, paracellular permeability appeared to be reduced in NCGS, which is associated with increased mRNA levels of claudin-4, a barrier-forming protein [68]. In contrast, Hollon et al. evaluated changes in transepithelial electrical resistance (TEER) after exposure to gliadin in tissue biopsies from celiac patients, NCGS, and non-celiac controls [72]. Although gliadin exposure reduced TEER in all groups, significant changes were observed when comparing active celiac or NCGS patients with celiac patients in remission, indicating that there is a barrier-altering effect exerted by gliadin in NCGS similar to celiac disease [72]. In vivo studies, using confocal endomicroscopy after intravenous fluorescein injection, confirmed a reduced barrier function in NCGS and reported small intestinal epithelial defects and luminal fluorescein loss after luminal wheat exposure, which was associated with increased expression of the pore-forming claudin-2 [73]. In a recent study, the serum levels of lipopolysaccharide (LPS)-binding proteins and antibody reactivity to microbial products were found to be elevated in patients with NCGS and correlate with circulating levels of intestinal fatty-acid-binding protein, suggesting a compromised gut barrier and microbial translocation [74]. Upon GFD treatment, the increase in markers of immune activation and epithelial cell damage changed significantly towards normalization in affected individuals, demonstrating a link between wheat-containing diet, a dysfunctional intestinal barrier, and systemic immune activation as underlying mechanistic components in NCGS [74]. In addition, intestinal dysbiosis seems to contribute to epithelial barrier dysfunction and associated inflammatory response to gluten, thereby contributing to the pathogenesis of NCGS, similarly to what has been shown in other gut disorders [75]. The presence of markers of systemic immune activation, inflammation, and gut epithelial cell damage in NCGS individuals, in the absence of celiac disease, provides a biological basis to explain both the intestinal and extra-intestinal manifestations of the gluten-related conditions.

### 3.2. Role of Wheat Components in NCGS Pathogenetic Mechanisms

In the NCGS framework, different subsets of patients may be sensitive to different cereal components. The main components suspected to trigger symptoms in NCGS are gluten, ATIs, and FODMAPs, either individually or in combination [76]. Gluten is a complex of various hydrophobic proteins, gliadins, and glutenins accounting for 80–85% of the total protein content of wheat [77]. Glutens contain a large amount of proline-rich polypeptide residues that make it resistant to proteolytic degradation in the gastro-intestinal tract [78]. When gluten proteins are consumed by susceptible individuals, a cascade of immune reactions is triggered, resulting in intestinal dysfunction and gluten-related syndromes [1]. However, the specific role of gluten as an NCGS trigger is still not fully understood. Gluten can interact with the intestinal epithelium through the C-X-C Motif Chemokine Receptor 3 (CXCR3), in this way promoting the release of zonulin by enterocytes and allowing the passage of molecules from the intestinal epithelium towards the lamina propria. Once gliadin peptides have entered the lamina propria, they could activate the innate immune system via TLR-2 and TLR-4 receptors, inducing the release of pro-inflammatory cytokines such as IP-10/CXCL10 and tumor necrosis factor (TNF) [79].

ATIs are a group of low-molecular weight proteins that are highly resistant to gastrointestinal proteases. Although the role of ATIs in NCGS is still uncertain, they have been proposed as molecules that have the potential to activate the innate immune system in NCGS [80]. Experimental evidence shows that wheat-derived ATIs exacerbate the intestinal immune response in humanized mouse models [81]. In vitro and in vivo studies have shown that ATIs can stimulate the innate immune system by activating TLR-4 on monocytes, macrophages, and dendritic cells in the intestinal mucosa and trigger an inflammatory reaction through activation of nuclear factor-κB (NF-κB) and subsequent production of pro-inflammatory cytokines and chemokines, including interleukin (IL)-8, TNF, or monocyte chemotactic protein-1 (MCP-1/CCL2) [82]. Meanwhile, the activated antigen-presenting cells migrate to peripheral lymph nodes and further amplify the ongoing immune response [82]. In the context of intestinal damage characterizing NCGS, an increased translocation of LPS and intact ATIs to the lamina propria could occur, triggering the release of pro-inflammatory mediators leading to a local and systemic inflammation [83].

FODMAPs are short-chain carbohydrates comprising fructans and galacto-oligosaccharides, found in many foods, including wheat [84]. The human body lacks enzymes to break down FODMAPs, which are slowly absorbed in the small intestine and pass undigested into the large intestine, where they are rapidly fermented by gut bacteria, producing gas and causing intestinal walls to stretch [85]. FODMAPs could trigger symptoms in different gastrointestinal disorders, including NCGS [86]. Biesiekierski et al. [87] observed that removal of dietary FODMAPs caused improvement in gastrointestinal symptoms in NCGS and irritable bowel syndrome. Additionally, NCGS patients have reported remission of symptoms following a low-FODMAP diet [87]. However, the specific role of cereal components, including gluten, ATIs, and FODMAPs, in the development of NCGS is still unknown. Further research is therefore needed to establish the exact role of dietary components in triggering symptoms in NCGS patients [88].

## 4. NCGS Dietary Management

The treatment of choice for NCGS management is a GFD, which improves the clinical manifestations both at intestinal and extra-intestinal levels. A GFD indicates the absence of gluten proteins from wheat or other cereals in natural as well as in processed foods [89]. The upper limit for gluten content in the GFD has been set at 20 ppm by the Codex Alimentarius Commission for International Food Standards of the United Nations [FAO]/World Health Organization. This cut-off limit is followed in many countries including Spain, Italy, the United Kingdom, Canada, and the United States. It is lowered to 10 ppm in Argentina and 3 ppm in other countries, such as Australia, New Zealand, and Chile [90]. Although a GFD is the only strategy for dealing with NCGS cases, it remains to be clarified whether a lifelong GFD is needed as in cases of celiac disease. A study by Carroccio et al. reported that 74% of NCGS patients continue to follow a wheat-free diet 8 years after diagnosis, and then wheat consumption may still cause symptoms [91]. Data indicate that patients with NCGS may have different gluten tolerance thresholds that should be evaluated to determine whether a strict GFD is needed [92]. Some authors have suggested that a new gluten re-challenge could be carried out in NCGS patients, after 1–2 years of GFD, by reintroducing the adequate dose of gluten tolerated [93]. On the other hand, a GFD is associated with an increased consumption of macronutrients, such as saturated fat and sugar, and lower intake of micronutrients such as iron, folic acid, and zinc than subjects following a regular diet [94]. In an observational study, patients with NCGS were shown to eat different foods than healthy individuals, with lower amounts of proteins, carbohydrates, fiber, and polyunsaturated fatty acids. Their diets should therefore be routinely analyzed and possibly corrected to avoid nutritional deficiencies [95]. Although a GFD efficiently reduces symptoms in most cases, some NCGS patients still report symptoms despite following a rigorous GFD for years after their diagnosis [96]. This evidence suggests that other components of wheat in addition to gluten may be responsible for symptoms in these NCGS patients. A low-FODMAP diet has also been found to be effective in reducing symptom score in NCGS patients [75]. However, the intake of this diet should be carefully considered as it has been associated with a low intake of natural antioxidants and micronutrients. Furthermore, FODMAPs can exhibit a prebiotic effect in colon bacteria, stimulating the growth of beneficial bacteria (lactobacilli and bifidobacteria) and limiting the colonization of harmful bacteria (*Bacteroides* spp., *Escherichia coli*, and *Clostridium* spp.) [75]. FODMAPs have also been shown to improve lipid metabolism, calcium absorption, and protective effects against colorectal cancer [88]. Therefore, dietary supplementation with prebiotics and vitamins is recommended in patients on a low-FODMAP diet [97]. Furthermore, a follow-up 4–6 weeks after starting a low-FODMAP diet is recommended to consider reintroducing high-FODMAP foods into the diet of NCGS patients [98]. In the dietary management of NCGS patients, the medical and dietitian advice should consider a GFD and a low-FODMAP diet only if an improvement in clinical manifestations is observed in order to prevent any nutritional deficiencies.

In an effort to meet the quality of life demands of patients with gluten-related diseases, much research has explored alternative strategies and therapeutic interventions to influence the gut response to gluten and potentially restore tolerance [99]. Growing interest is aimed at identifying healthier eating habits (rather than a GFD) or consuming specific foods to contribute to the much-desired relief of NCGS symptoms. Observational and interventional evidence are now available on the benefits of consuming plant-based foods and beverages. High consumption of fruits, vegetables, and minimally processed grains promote wellbeing and longevity. Plant-based food products are unique and rich sources of a wide variety of bioactive phytochemicals [100]. Among these, polyphenols have attracted considerable attention in recent years for their health-promoting properties in reducing the risk of cardiometabolic and neurodegenerative diseases [101,102]. Increasing research on polyphenols is now uncovering the functional and therapeutic potential of these natural compounds in various disease contexts, including gluten-related diseases.

## 5. Polyphenols as Tools for Mitigating NCGS Symptoms

We here provide an up-to-date revision of the effects of polyphenols as possible dietary strategies to counteract the toxic effects of gluten, resulting in improved quality of life of patients with gluten-related intestinal diseases. In the attempt to explore the molecular and cellular mechanisms underlying the therapeutic and preventive properties of polyphenols in gluten-related disorders, we address different aspects including the metabolic fate of polyphenols as well as the potential polyphenol targets such as gluten seizure, intestinal epithelial barrier disruption, immune response, oxidative stress, inflammation, and dysbiosis.

### 5.1. Polyphenol Bioavailability

**Figure 2:**
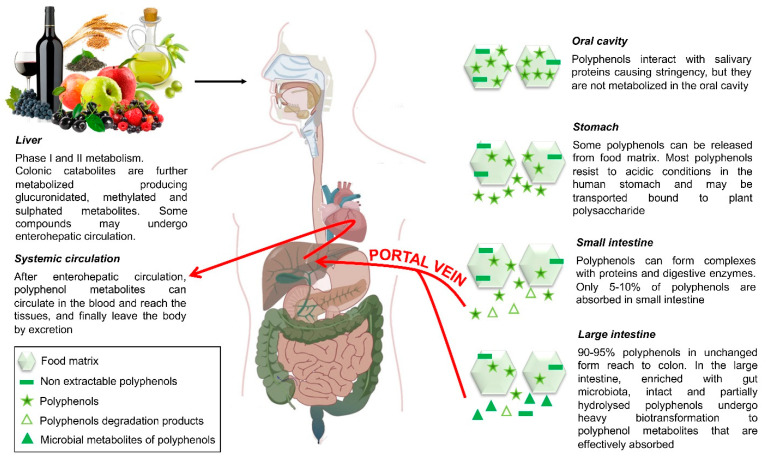
Schematic representation of dietary polyphenols’ fate along the digestive tract.

Polyphenols, the most common phytochemical bioactive components in our diet, are plant secondary metabolites derived from the shikimate/phenylpropanoid pathway [103]. Until now, more than 8000 polyphenols have been identified in nature, usually divided into different classes according to the number of phenolic rings that they contain and the structural elements that link these rings to one another. They occur in a non-conjugated form (aglycone) or conjugated with sugars, carboxylic and organic acids, amines, lipids, and other phenols. The distribution of polyphenols in plant-based foods and beverages is not uniform, being that their content is dependent on numerous genetic, environmental, and technological factors. Moreover, the intake of polyphenols varies by geography, possibly due to different diet habits [104]; however, the average dietary intake of polyphenols is speculated to be about 1 g/day. Typically, after ingestion of polyphenols, the absorption of some but not all compounds occurs in the small intestine [100]. Although some flavonoids, such as quercetin and anthocyanins, can also be absorbed at the gastric level, most glycoside polyphenols resist acid hydrolysis in the stomach and arrive intact in the intestine, where they are preferentially absorbed [100]. There, conjugated glycosides are hydrolyzed by intestinal epithelial cells and undergo some phase I (oxidation, reduction, and hydrolysis) and phase II (conjugation) reactions including methylation, sulfation, and glucuronidation [105]. Several factors can affect the absorption, metabolism, and distribution of polyphenols in humans. These include polyphenol-related factors such as the chemical structure (aglicone) and type of sugar in the glycoside, as well as their interaction with food proteins, carbohydrates, and fiber [106]. Polyphenols that are not absorbed in the small intestine constitute a large fraction (90–95% of total polyphenol intake) and may accumulate in the large intestine up to the millimolar range, where most of them are extensively metabolized by the intestinal microbiota (Figure 2) [107]. Current investigations on dietary polyphenols mainly focus on a fraction of polyphenols, known as extractable polyphenols, so-called as they can be extracted from food with aqueous–organic solvents (e.g., water, methanol, ethanol, acetone) [108]. However, another fraction of polyphenols remains in the residues after solvent extraction, and they are known as “non-extractable” polyphenols [109]. They are mainly polyphenols bound to dietary fiber or other macromolecules through hydrophobic interactions, hydrogen bonding, and covalent bounding [109]. The “non-extractable” polyphenols include both high-molecular-weight polymeric polyphenols and low-molecular-weight phenolics attached to macromolecules. With regards to their chemical nature, they comprise mainly polyphenols such as proanthocyanidins, other flavonoids, phenolic acids, and hydrolysable tannins [110]. Non-extractable polyphenols are not released in significant quantities from the food matrix during digestion in the stomach or small intestine; therefore, they reach the colon nearly intact [111]. In the colon, “non-extractable” polyphenols may be released from the food matrix by the action of microbiota and become bioavailable and bioactive, giving rise to small phenolic acids and aromatic catabolites (Figure 2) [111]. The better colonic bioavailability of “non-extractable” polyphenols than extractable polyphenols makes them promising candidates for promoting colon health. Recent studies demonstrate “non-extractable” polyphenol metabolites persist in human plasma for extended times up to 3–4 days after intake with significant concentrations. The absorption of these catabolites occurs slowly and continuously through the colon, leading to sustained and continuous increases in their concentrations rather than plasma peaks [112]. These microbial by-products can then be absorbed and further metabolized in the liver before being excreted into the urine (Figure 2). Unabsorbed metabolites are eliminated through the stool. Preclinical studies with these metabolites at the concentrations that can be reached in plasma have reported anti-inflammatory and antioxidant effects, as well as gut barrier protection and dysbiosis, that could be related to health benefits observed in vivo after the intake of the non-extractable polyphenols, as described below.

### 5.2. Role of Polyphenols in Gluten Binding and Capture

**Figure 3:**
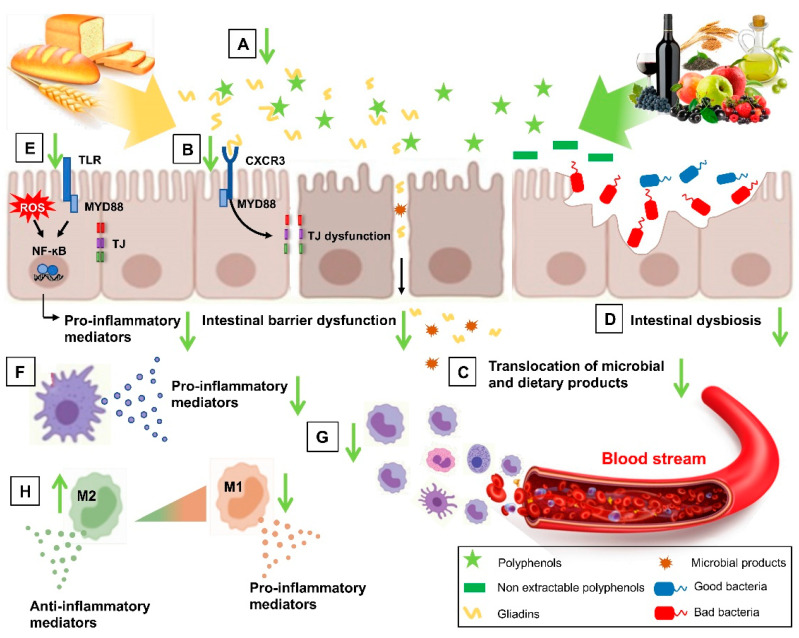
The potential targets of polyphenols and their metabolites in NCGS. Polyphenols can bind and sequester gluten, reducing gluten bioavailability (**A**); they can inhibit the interaction between gliadin peptides and CXCR3 receptors, restoring the altered intestinal permeability (**B**) then reducing the translocation of microbial and dietary products (**C**); they can also affect the intestinal dysbiosis improving the intestinal barrier dysfunction (**D**). Polyphenols can blunt oxidative stress and inflammation-induced pathways at gut level (**E**) and suppress the overwhelming inflammatory immune response (**F**), reducing the recruitment of myeloid cells in the lamina propria (**G**) and increasing M2-polarized anti-inflammatory macrophages (**H**). The green arrow indicates the effects of polyphenols: up arrow corresponds to induction; down arrow corresponds to inhibition. TLR: Toll-like receptor; CXCR3: C-X-C Motif Chemokine Receptor 3; TJ: tight junction; ROS: reactive oxygen species; NF-ĸB: nuclear factor-κB; M1: M1-polarized macrophage; M2: M2-polarized macrophage.

Several studies show that polyphenols can exert health-promoting properties by interacting with other macromolecules such as proteins [113]. Polyphenols have been shown to bind preferentially to proteins rich in proline, such as gluten proteins [114,115]. The formation of gliadin–polyphenol complexes can be beneficial in protecting against gluten-related disease if these complexes allow sequestration of the protein and prevent it from interacting with gut milieu. In fact, the inflammation persists only until the gluten peptides are free to reach the intestinal mucosa. Physical sequestration of gliadins by complexation has been shown to be effective in clinical and animal studies with a synthetic and non-bioavailable polymer (BL-7010), which was able to precipitate gliadins from the solution, forming insoluble complexes and preventing an immune response in gluten-sensitive mice [116]. A similar seizure effect has been demonstrated by Van Buiten in vitro using green tea polyphenols as biological sequestrants [117]. In vitro digestion studies have shown that green tea polyphenols are capable of precipitating both native and pepsin/trypsin-digested gliadins through the formation of insoluble complexes [117]. This complexion can make gliadin inaccessible to the body and thus prevent absorption and a subsequent immune response. The ability of polyphenols to precipitate gliadins is of considerable interest for therapeutic development, as this could potentially allow “scavenging” of gliadins by polyphenols during the digestive process (Figure 3). Interactions between gliadins and epigallocatechin-3-gallate (EGCG) were also preserved at various pH (at pH 2.0, 6.8, and 7.2), demonstrating a similar formation of insoluble complexes and interactions between EGCG and specific residues of proline and glutamine [118]. Based on the polyphenols’ ability to interact with gliadins and mask epitopes, polyphenol-rich extracts from artichoke leaves, cranberries, apples, and green tea leaves modulated the immunogenicity and allergenicity of gluten proteins [119]. Previous reports have also demonstrated the ability of grape seed procyanidins as well as quercetin and anthocyanins to bind and structurally modify gliadin proteins, potentially decreasing their immunoreactivity [120]. Some studies have evaluated the biological implications of interactions between gliadin proteins and peptides with polyphenols using an in vitro model of human intestinal epithelial cells (Caco-2). They represent a simplified model of the intestinal barrier in which human colonocytes are grown on a semi-permeable membrane until confluence takes place. This cell model expresses the characteristics of the human small intestine, including the enzymes of the brush edge, the proteins of the tight junction (TJ), and the microvilli, and has been widely used to study the mechanisms and signaling pathways involved in intestinal diseases including gluten-related diseases [121]. In Caco-2 cell model, EGCG reduced the transepithelial transport of the bioactive gluten peptide [122,123], highlighting the ability of dietary doses of polyphenols to effectively eliminate bioactive gluten peptide under physiological conditions, leading to a significant reduction in the concentration of free peptide in the basolateral compartment. Similarly, procyanidins were able to reduce the apical–basolateral translocation of gluten peptides in Caco-2 [123,124]. These findings suggest that polyphenols, through the sequestration of gluten proteins, may became a potentially new therapeutic approach to prevent the onset of gluten-related responses and associated symptoms.

### 5.3. Antioxidant and Anti-Inflammatory Effect of Polyphenols at Gut Level

Gluten-related disorders are characterized by chronic gut inflammation with increased secretion of inflammatory cytokines. This condition in turn can alter intestinal permeability and produce large quantities of reactive oxygen species (ROS), modifying the cellular redox state and leading to reduced antioxidant capacity [125]. The imbalance between ROS production and cell antioxidant defenses in favor of oxidants is referred to as oxidative stress and can lead to cell damage. Impaired redox balance has been shown to cause severe damage to different cell components, including proteins, lipids, and DNA. Several studies have shown that gluten exposure causes an intracellular oxidative imbalance, characterized by an increase in the levels of lipid peroxidation products and in the oxidized/reduced glutathione ratio, as well as a reduction in the number of sulfhydryl groups bound to proteins [126,127]. NADPH oxidases (NOX) are a group of different enzymes collectively involved in the production of ROS, in particular superoxide, and creating a respiratory burst, which plays a crucial role in the innate immune response to pathogens and inflammatory and autoimmune diseases [128]. NOX activity and expression have been reported to significantly increase under gliadin-induced stress conditions [129]. Mitochondria also play a crucial role in oxidative stress, and mitochondrial abnormalities have been demonstrated in various inflammatory bowel diseases. Therefore, antioxidant treatment strategies targeting mitochondria have been proposed [130,131]. The molecular mechanism linking gluten toxicity with mitochondria, ROS, and inflammation could rely on the ability of mitochondrial ROS to activate the caspase-1-activating multiprotein complex (NLRP3 inflammasome), as found in the peripheral blood mononuclear cells from celiac patients treated with gliadin [132]. In Caco-2 cells, pepsin-trypsin-digested gliadin induces oxidative stress, leading to a mitochondrial response consisting of the compensatory induction of biogenesis [133]. The effects of gliadin peptides are counteracted by pre-treatment of Caco-2 cells with the synthetic phenolic compound butylated hydroxytoluene (2,6-di-tbutyl-p-cresol), which exerts its antioxidant activity as an ROS scavenger [133]. Alteration in oxidative balance induced by gliadin peptides in intestinal cells is involved in the activation of transcription factor NF-κB. NF-κB activation induces the transcription of pro-inflammatory cytokines and enzymes such as cyclooxygenase-2 (COX-2), cytosolic phospholipase A2 (cPLA2), and inducible nitric-oxide synthase (iNOS) with a consequently higher production of prostaglandins and nitric oxide metabolites contributing to the oxidative stress [121]. In addition, it has been reported that levels of proinflammatory cytokines such as INF-γ, IL-1β, TNF, IL-6, and IL-8 in the plasma of celiac patients were significantly higher compared to non-celiac subjects [134]. The increased oxidative stress is also involved in the downregulation of PPARγ mediated by tissue transglutaminase, which appears to be increased in the celiac mucosa and in turn may contribute to NF-κB activation. Polyphenols have historically been recognized as antioxidants, reducing agents, and scavengers of free radicals. It is now known that polyphenols, as well as their metabolites, can modulate transcription events of genes involved in oxidative metabolism, including the enzymes producing ROS, such as xanthine oxidase, NOX, and iNOS, as well as enzymes involved in the metabolism of arachidonic acid such as PLA2, COX, and lipoxygenase [135]. Moreover, polyphenols are able to upregulate detoxifying enzymes/phase II antioxidants, such as emeoxygenase-1, glutathione peroxidase, glutathione-S-transferase, catalase, and superoxide dismutase (SOD), by targeting the common nuclear factor erythroid 2 related to factor 2 (Nrf2) [136]. There is now an emerging acceptance that polyphenols, as well as their metabolites, affect inflammatory response and modulate the transcription of inflammatory genes by inhibiting the activation of the redox-sensitive transcription factor NF-κB, the main pathway driving inflammatory cascades, as well as the upstream MAPK and signal transduction pathways [137]. Polyphenols also induce overexpression of Sirtuin 1 in a variety of models, thus helping to protect cells from oxidative stress, inflammation, and mitochondrial dysfunction by deacetylating transcription factors and proteins, such as NF-κB, forkhead box class O3, and co-activator PPAR-γ 1α (PGC-1α) [138].

These results demonstrated that polyphenols can improve gut health, even in the framework of gluten-related diseases, by preserving intestinal cells against oxidative aggression and inflammation triggered by gluten proteins (Figure 3) [139].

A recent study by Gupta et al. [140] reported that gliadin promotes disease pathogenesis by inducing inflammation and cellular damage, which further alter the cellular homeostasis of intestinal cells. The pre-treatment of curcumin counteracts the adverse effect of gliadin and protects the intestinal cells via reducing the inflammation and helping the cell to regain cellular morphology [140]. In vivo and in vitro studies have suggested that green tea and its major polyphenolic component, EGCG, may mitigate damage to the small intestinal mucosa and reduce production of inflammatory cytokines, decreasing oxidative stress in epithelial cells [117,141]. In this context, our recent study also highlighted the gut-protective effects of grape pomace polyphenolic extract in dumping the overwhelming inflammatory response induced by LPS and TNF in intestinal epithelial cells [142]. These effects occur by preventing the intestinal expression of multiple inflammatory markers including IL-6, CCL2, and matrix metalloproteinases (MMPs), which was mediated by the inhibition of NF-ĸB and the reduction of intracellular ROS levels. Furthermore, in intestinal epithelial/endothelial cells’ co-culture systems, transepithelial grape pomace polyphenolic extract suppressed the endothelial expression of pro-inflammatory markers, leading to a better vascular endothelial function [142]. Similarly, in this co-culture model, kaempferol [143] and anthocyanins from purple carrots and potatoes [144], as well as the anthocyanidin cyanidin-3 glucoside [145], mediated anti-inflammatory effects by targeting the NF-κB pathway, consequently resulting in lower levels of pro-inflammatory cytokines. Moreover, stilbenes, catechins, and epicatechins, as well as quercetin, exhibited intestinal anti-inflammatory properties by inhibiting the activity of NF-ĸB and reducing the production of several proinflammatory mediators [146]. In a co-culture model, the pre-treatment with curcumin decreased the gene expression of pro-inflammatory cytokines such as TNF and IL-6 production and significantly improved the intestinal barrier through an upregulation of gene expression levels of various TJ proteins [147]. Collectively, several findings showed that dietary polyphenols exert antioxidant and anti-inflammatory roles and have a protective effect on intestinal epithelium; therefore, their adoption could contribute to preserving gut health and protecting against the toxicity of gliadin peptides in gluten-sensitive subjects. Most studies are focused on foods and beverages in which phenolic antioxidants may be easily released from the food matrix, reaching a peak in plasma antioxidant capacity 1–2 h after the intake. However, plant foods contain significant amounts of “non-extractable” polyphenols associated with dietary fiber, whose absorption occurs slowly and continuously through the colon, leading to sustained and continuous increases in their concentrations rather than plasma peaks [112]. In vivo studies have shown that the intake of polyphenols bound to dietary fiber from grape seeds enhanced the antioxidant status in the large intestine in rats [148]. In association with the antioxidant capacity of “non-extractable” polyphenols, an increase in the expression of endogenous antioxidant systems has been shown, thus counteracting overproduction of intestinal ROS and exhibiting a beneficial effect on gastrointestinal health. Animal studies also showed that the intake of grape antioxidant dietary fiber reduced apoptosis and induced a pro-reducing shift in the glutathione redox state of the rat proximal colonic mucosa [149]. In healthy volunteers, an acute intake of 15 g of a dietary fiber rich in associated phenolic antioxidants increased the antioxidant capacity of plasma in comparison to a control group, becoming significant 8 h after the intake. This shows that phenolic antioxidants associated with dietary fiber are at least partially bioavailable in humans and contribute to the antioxidant capacity of the gut [150], suggesting their role in preserving gut health by reducing oxidative stress. Finally, both in vitro and in vivo, quercetin exposure was able to induce secretory leukocyte protease inhibitor (Slpi) [151], a member of the innate immunity-associated protein family, whose main function appears to be tissue protection against the deleterious consequences of prolonged inflammation [152]. In particular, Slpi expression was detected in mucosal resident dendritic cells in mice receiving a quercetin-enriched diet [153] and in intestinal organoids exposed to quercetin-enriched culture media [154]. The overall idea is that the presence of polyphenols in the intestinal lumen may favor inflammatory suppression and the tissue repair pathway able to prevent the initiation of acute inflammatory responses.

### 5.4. Immunomodulatory Role of Polyphenols

Taking into consideration the role of inflammation and immune deregulation in the pathogenesis of gluten-related disorders, the immunomodulatory properties of dietary polyphenols, as demonstrated by reports in model systems and human trials, provide potential mechanisms for polyphenol benefit in NCGS. Examples are the observations that polyphenols from different food sources, including olive oil, red grape/wine, fruits, vegetables, cereals, cocoa, coffee, and tea, exert immunomodulatory effects, either stimulatory or inhibitory depending on the context, in both the innate and adaptive immune branches and in particular in monocytes/macrophages, T and B cells, dendritic cells, natural killer (NK) cells, and on T cell differentiation, with associated benefits in immune-related pathologies such as inflammatory bowel disease, autoimmune diseases, atherosclerosis, and cancer [155]. Several in vitro reports have shown the ability of polyphenols to dampen the inflammatory activation of monocytes and the corresponding expression and release of humoral factors, such as matrix-degrading (MMPs) and inflammatory (COX-2) enzymes, cytokines (TNF, IL-1β), and chemokines (CCL2, M-CSF, CXCL-10) [46,156,157]. Interestingly, polyphenols such as lycopene, tyrosol, and quercetin inhibited the inflammatory response to gliadin plus IFN-γ in murine macrophages by reducing iNOS and COX-2 gene expression via the inhibition of NF-κB, interferon regulatory factor-1 (IRF-1), and signal transducer and activator of transcription-1alpha (STAT-1alpha) [158]. Different polyphenols, including resveratrol, quercetin, or those from cocoa extracts and green tea, suppressed the inflammatory response in macrophage, myeloid, and plasmacytoid dendritic cells [159,160] and macrophage polarization toward the inflammatory M1 phenotype, while promoting macrophage metabolism by oxidative pathways and increasing M2-polarized macrophages [161], which was linked to the prevention of tissue and systemic inflammation in vivo (Figure 3) [162,163]. Other in vitro and in vivo studies reported significant stimulating effects by polyphenols on Treg cells [162,163,164,165,166], thus promoting immune tolerance and suppressing autoimmunity. In an animal model of rheumatoid arthritis, an autoimmune disease, besides increasing Treg levels, a grape seed proanthocyanidin extract reduced inflammation by upregulating the number of Th2 cytokine-producing cells and decreasing the levels of pro-inflammatory Th1 cytokines [165]. Similarly, tea polyphenols modulated CD4+ T cell differentiation by reducing Th1, Th9, and Th17 differentiation, the levels of IL-6 and its soluble receptor (IL-6R), and prevented the suppression of Treg cell development, thus improving the Th17/Treg balance and the autoimmune response [167]. Some studies also found that polyphenols increased the number and/or cytotoxic activity of NK cells [168].

An important role of polyphenols is the regulation of pattern recognition receptors, including TLRs, and the consequent inflammatory response, as demonstrated for resveratrol, the result of which being capable of inhibiting the expression levels of TLR2 and TLR4 [162], and the signal transduction of TLR3 and TLR4, by inhibiting TANK binding kinase 1 and the consequent NF-κB activation and COX-2 expression [169]. Resveratrol-treated mice showed a relief of inflammatory bowel disease symptoms, which was associated with increased expression levels of anti-inflammatory cytokines (IL-10) and decreased expression levels of pro-inflammatory cytokines (TNF, IL-1β, IL-8, IL-6) in both colon and spleen tissues [170]. These effects were also related to reduced expression and activation of pro-inflammatory signaling SUMO1 and β-catenin in the human colonic epithelial cell line [170]. Similar results were reported for other polyphenols in the mouse model of inflammatory bowel disease [171,172]. An alleviation of symptoms of inflammatory bowel disease accompanied by reduced systemic inflammatory cytokines has been observed after supplementation with a polyphenol extract (gallotannins and gallic acid) from mango in patients with mild-to-moderate inflammatory bowel disease [173]. In patients with ulcerative colitis, oral administration of curcumin capsules in combination with a standard therapy was superior to the standard therapy alone in inducing disease remission [174]. In a mouse model of ulcerative colitis, resveratrol reduced colonic inflammatory cell infiltration, erosion, and edema, as well as plasma and intestinal mucosal cytokine levels including IL-10, TGF-β1, IL-6, and IL-17, and decreased the number of Th17 cells while increasing the number of Treg cells in the spleen [175]. Collectively, the immunomodulatory properties described here and widely reported in the literature [176,177] point to a potential therapeutic effect of polyphenols also on gluten-related disease outcomes, although further specific preclinical and clinical investigations are required. Even if the relative percentage of microbial species in the duodenum is relatively low compared to the ileum and the colon, part of the immunomodulatory abilities of the polyphenols may be mediated by a favorable regulation of the gut microbiota, which is crucial for immune system development and function and for intestinal homeostasis (see Section 5.6).

### 5.5. Role of Polyphenols in Intestinal Epithelial Barrier

The intestinal epithelium is a unicellular layer, which constitutes the largest and most important barrier against the external environment. It acts as a selectively permeable barrier, allowing the absorption of nutrients, electrolytes, and water through specific membrane transporters or channels (transcellular pathway), while maintaining an effective defense against intraluminal toxins, antigens, and enteric flora through paracellular pathways consisting of a complex system of junctions crucial for the transport between adjacent cells [178]. The gut barrier is composed of several physical and chemical components. Intestinal epithelial cells display densely packed microvilli joined at their apical side by TJ proteins including zonulin, occludin, and claudins. This monolayer is renewed constantly and is covered by a protective mucus layer that is impregnated with several immune and antimicrobial factors produced by the host.

Maintaining the integrity of the gut barrier is crucial and avoids structural and functional changes of the intestine that can lead to various disorders. Impairment of the epithelial barrier has been described in all gluten-related diseases, but its role as a potential pathogenetic co-factor is still under investigation. Similar to other gluten-related diseases, NCGS is characterized by increased intestinal permeability and disassembly of epithelial TJ, with an impaired barrier function that favors the non-selective diffusion of gluten-derived peptides and activates immune reactions in response to gluten [179]. In differentiated Caco-2 cells, gliadin peptides have been shown to trigger a reorganization of actin filaments and to alter the expression of occludin, claudin-3, claudin-4, zonulin, and E-cadherin proteins. In addition, gliadin peptides can bind to the CXCR3 chemokine receptor expressed on the surface of intestinal epithelial cells. In response, a cascade of intracellular signals is activated, leading to the release of zonulin in a MyD88-dependent manner, resulting in disassembly of PCK-α-dependent TJ and increased permeability [180].

Since growing evidence suggested a direct role of the intestinal barrier in the pathogenesis of gluten-related disorders, efforts have been made to identify the components of the epithelial barrier as possible therapeutic targets. So far, the most widely studied therapeutic target within the intestinal epithelial barrier is zonulin [180]. Gliadin, in fact, can activate zonulin signaling and alter intestinal permeability. A zonulin inhibitor, larazotide acetate, has been tested for its ability to reverse the increased intestinal permeability caused by zonulin signaling [181]. In vitro experiments have shown that larazotide acetate is capable of inhibiting TJ rearrangements, thus preventing increased epithelial permeability triggered by exposure to gliadin and proinflammatory cytokines [181]. Some human studies have subsequently confirmed the protective effect of zonulin inhibition, resulting in improvement of gluten-induced symptoms [182,183].

To date, no other molecule with an effect on intestinal barrier function has been tested as a potential therapeutic option for gluten-related disorders. In recent decades, polyphenols and their metabolites have gained attention for their promising beneficial health effects, showing a role in the regulation of the intestinal barrier [184]. At present, the exact mechanisms linking polyphenols with intestinal epithelial barrier function have not yet been established [184]. Some studies have hypothesized that polyphenols may improve barrier function by regulating oxidative stress through ROS downregulation by targeting different members of the NF-κB pathway or by antagonizing its activation. Pro-inflammatory cytokines have been shown to activate NF-κB and alter epithelial barrier function by disassembling TJ. Conversely, polyphenols have been documented to block NF-κB activation by inhibiting IκB kinase phosphorylation and/or preventing IκB proteasomal degradation. Other important factors include multiple protein kinases, such as MAPK, phosphoinositide-3-kinase/Akt, PKC, tyrosine kinase, MLCK, and adenosine monophosphate (AMP)-activated protein kinase (AMPK), which regulate TJ expression in epithelial cells and intestinal barrier function.

Some polyphenols such as quercetin, curcumin, EGCG, and myricetin have been shown to improve epithelial barrier function through inhibition of several kinases involved in phosphorylation of target proteins that control the intestinal permeability [185]. By interfering with this signaling, polyphenols prevent the disassembly of TJ proteins and restore barrier integrity (Figure 3). Polyphenols also reinforce gut barrier function and morphology through the maintenance of the epithelial mucus layer in different mouse models of a defective gut epithelium [186]. The main lines of evidence on the effects of polyphenols in modulating the potential mediators and regulatory pathways involved in intestinal barrier are derived from in vitro studies performed mainly using the Caco-2 cell line as a model of intestinal barrier function. The main protective evidence is available for extracts rich in polyphenols, or pure polyphenols, including flavonols, stilbenes, procyanidins, and anthocyanins. In differentiated Caco-2 cells, they increase TEER in conjunction with the expression of numerous tight-junction proteins such as zonulin proteins, occludin, and claudin and the inhibition of several protein kinases that regulate intestinal permeability [187,188,189,190,191].

The effects of polyphenols and polyphenol-rich extracts in modulating intestinal barrier function have also been confirmed in animal models, including rat models fed diets high in fat, mannitol, or treated with inflammatory cytokines or chemicals [192]. Polyphenols from different dietary sources including berberine, epicatechin, and anthocyanins, as well as extracts rich in polyphenols, exhibited gut-protective effects by increasing the expression of TJ proteins and by modulating the key genes involved in the inflammatory process, e.g., AMPK, ERK, and NF-κB [193,194,195,196,197,198,199,200,201]. Consistent with the observations reported in vitro, the tested compounds were shown to increase the expression of zonulin, occludin, and several claudins involved in the functioning of TJ. Regarding human studies, recent literature suggests that polyphenols can modulate intestinal barrier function through a range of direct and indirect effects, including impact on the gut ecosystem and the immune system [184]. Future research should be aimed at identifying polyphenols and/or their metabolites possibly involved in the modulation of intestinal barrier function, also demonstrating their specific dose-dependent mechanisms of action.

From the available data, the activity of polyphenols seems plausibly to be a consequence of multiple mechanisms, which may also depend on the type and quantity of the compounds considered. In particular, “non-extractable” polyphenols bound to fibers seem to have a greater effect on the function of the intestinal barrier than polyphenols or fibers alone (Figure 3) [202]. Fractions rich in non-extractable polyphenols and fiber from grape peel powder have been shown to be good alternatives to protect the barrier function and colonic injury in a colitis model in rats. The non-extractable polyphenols-rich fraction decreased the extension of colonic lesion and reduced claudin-2, a marker of barrier permeability predominantly expressed in leaky gut epithelia, whereas the fraction that was composed mostly of fiber had no effect. The downregulation of claudin-2 may be in part due to the anti-inflammatory effect of polyphenols, since some pro-inflammatory cytokines such as TNF and IL-6 have been shown to be involved in the upregulation of claudin-2 expression and increase of the intestinal permeability. Moreover, the polyphenols associated with dietary fiber appeared to play a remarkable protective role, as it was the only fraction to reduce the area of colonic lesion. Grape peel polyphenols-rich fractions or fiber increased the expression of zonulin or occludin in the colon tissue. Similar results have been reported for cocoa powder and cocoa fiber (poor in polyphenols), which are mostly composed of insoluble dietary fiber and were more effective than inulin (a soluble and highly fermentable fiber) for increasing occludin expression in the colon tissue [203]. Further studies on the mechanisms responsible for the protective effects of dietary polyphenols and their interactions with the food matrix, including dietary fiber, are needed, contributing to the identification of the most suitable type of polyphenol or fiber for the diet of patients with intestinal diseases, such as gluten-related disorders associated with defects in barrier function.

### 5.6. Role of Polyphenols in Intestinal Dysbiosis in Gluten-Related Disorders

Recent evidence, including a few studies on human intervention, have reinforced the idea that, in vivo, polyphenols modulate the function of intestinal cells through a series of direct and indirect effects, including their impact on the intestinal microbial community (Figure 3) [184]. The following will describe the involvement of the gut microbiota in gluten-related disease and in particular in NCGS and the role and mechanisms by which dietary polyphenols affect intestinal dysbiosis.

#### 5.6.1. Gut Microbiota and Gluten-Related Disease

The human gut microbiota is the largest complex and dynamic micro-ecosystem in the body, residing in the gastrointestinal tract, mostly in the large intestine, and consisting of trillions of microorganisms interacting with each other and with the human host, including mainly bacteria but also fungi, archaea, and viruses [204]. Gut microbiota plays a crucial role in host physiology, so that its alteration in composition and/or function, i.e., dysbiosis, is involved in the onset and progression of gastrointestinal diseases, cardiometabolic diseases, cancer, cognition, and neurodegenerative disorders [205]. The gut microbiota is therefore recognized as a potential relevant therapeutic target for many chronic diseases. Although the gut microbiota shows high interindividual variability and changes throughout an entire life under the influence of endogenous and exogenous factors, about 90% of the gut bacteria is represented by the phyla Bacteroidetes (including genera *Bacteroides* and *Prevotella*) and Firmicutes (encompassing genera *Lactobacillus*, *Bacillus*, *Clostridium*, *Enterococcus*, *Ruminococcus*, *Eubacterium*, *Faecalibacterium*, and *Roseburia*), followed in much lower abundance by Actinobacteria (namely *Bifidobacterium* and *Proteobacteria*), and Verrucomicrobia (namely *Akkermansia* spp.) [206].

Gut microbiota essential functions encompass the maintenance of the structural integrity of the epithelial barrier, the modulation of the development and function of the immune system, antimicrobial protection, the metabolism of carbohydrates, lipids, and proteins, and the synthesis of several vitamins and neurotransmitters. The metabolic activity of the intestinal bacteria includes the biotransformation of bile and xenobiotics and the production of a vast array of metabolites, which exert important local and systemic effects [207]. These include short-chain fatty acids (SCFA, mainly acetate, propionate, and butyrate) deriving from the anaerobic fermentation of indigestible polysaccharides, such as dietary fiber and resistant starch, mostly performed by the Firmicutes and Bacteroidetes phyla: SCFA have been regarded as beneficial metabolites capable of reinforcing the intestinal mucosal barrier, suppressing intestinal and extra-intestinal inflammation, regulating immune signaling, and influencing energy homeostasis, fat expansion, and insulin response. Furthermore, harmful metabolites can be produced by the gut microbiota, such as several secondary bile acids, p-cresol, p-tyramine, and trimethylamine-N-oxide [207]. The relationship between gut microbiota and gluten-related disorders is complex and still largely unknown, with reported contradictory findings regarding the role of gut microbiota in the risk, pathogenesis, and clinical manifestations of gluten-related disorders. Some evidence regarding celiac disease converges to the observation of an imbalance in the microbiota composition and diversity in active celiac disease, with decreased abundance of *Lactobacillus* and *Bifidobacterium* and increased abundance of *Bacteroides*, *Staphylococcus*, and *E. coli* [208,209]. Furthermore, celiac disease-associated fecal bacteria or fecal bacteria stimulated with gliadin demonstrated increased virulence and pro-inflammatory effects in immune and intestinal epithelial cells [210,211]. The observed dysbiosis is attenuated but not completely restored after a GFD [212,213], and the celiac disease-associated dysbiosis has been shown to associate with the persistent gastrointestinal symptoms in patients treated with a GFD [214]. A recent evaluation of the intestinal microbiota composition and functions in patients with celiac disease and NCGS found that the genus *Actinobacillus* in duodenal biopsies and the *Ruminococcaceae* family in fecal samples were higher in patients with NCGS, while *Novispirillum* was higher in the duodenum of patients with celiac disease [215]. Notably, an unexpected and increased abundance of duodenal *Pseudomonas* was reported after 4 weeks of consumption of a GFD in the NCGS group [215].

However, further clarifications are required because other reports show no such differences in gut microbiota [216], and it is still not clear if intestinal dysbiosis is the cause or effect of gluten-related disorders. Probiotics have been shown to reduce the inflammatory response and to restore normal proportions of beneficial bacteria in the gastrointestinal tract, but their use in treating gluten-related diseases needs further studies [217]. Interestingly, a higher proportion of Firmicutes (*Clostridium* spp.) and Proteobacteria (*Enterobacteriaceae*) and lower proportions of Actinobacteria (including the genus *Bifidobacterium*) have been found in infants with a high genetic risk for celiac disease (HLA-DQ2) compared with those with a low genetic risk (non-HLA-DQ2/8 carriers) [218]. This suggests that host genotype may select the bacteria colonization that may contribute to confer the risk of celiac disease. As demonstrated by studies in germ-free mice, the gut microbiota is crucial for the development of a fully functioning immune system, including the development of secondary lymphoid tissue, Th cell differentiation, and regulatory T cells [219]. Alterations in microbial composition or colonization may impact intestinal homeostasis and immune responses to food antigens, leading to food intolerance or sensitivities [219]. Furthermore, different proteolytic bacteria, mainly belonging to the Firmicutes phylum but also including *Bifidobacterium* and *Bacteroides fragilis*, can metabolize partially digested gluten peptides present in the intestinal lumen, resulting in different patterns of gluten degradation, which can increase or decrease the immunogenicity of gluten peptides [220]. Interestingly, gluten metabolized by opportunistic bacteria such as *Pseudomonas aeruginosa*, commonly found in patients with celiac disease, may activate the gluten-specific T cells of patients with celiac disease, while the degradation of gluten peptides by *Lactobacillus* spp. obtained from healthy individuals without celiac disease participates in reducing their immunogenicity [221].

#### 5.6.2. Polyphenols and Gut Microbiota

Diet plays a predominant role in modulating the composition and function of the gut microbiota [222]. Among the dietary compounds mostly affecting the gut microbiota, polyphenols have been shown to act through a direct antimicrobial effect and a direct beneficial bacterial stimulatory effect. Indeed, on the one hand, polyphenols can selectively inhibit the development of potential pathogenic species often associated with intestinal and metabolic disorders [223,224,225,226]. Furthermore, by acting as prebiotic substrates, polyphenols stimulate the growth and the activity of beneficial bacterial species, such as *Akkermansia muciniphila*, *Bacteroides thetaiotaomicron*, *Faecalibacterium prausnitzii*, *Bifidobacteria*, and *Lactobacilli*, thus promoting ecological shifts [186,227,228,229]. In metabolic syndrome patients, red wine polyphenols have been demonstrated to increase the abundance of fecal *Bifidobacteria* and *Lactobacillus* (beneficial for the intestinal barrier) and butyrate-producing bacteria (*Faecalibacterium prausnitzii* and *Roseburia*), while decreasing the abundance of pathogenic bacteria such as LPS producers (*Escherichia coli and Enterobacter cloacae*) [230]. As such, the antimicrobial action of polyphenols may be opportunistically used by other species to occupy freed ecological niches. Grape polyphenols have been shown to counteract intestinal and systemic inflammation, intestinal barrier dysfunction, and endotoxemia in high fat diet-fed obese mice, along with improvements in metabolic outcomes and changes in gut microbiota, as manifested by an increase in *Akkermansia muciniphila*, important for gut homeostasis, and decreased Firmicutes-to-Bacteroidetes ratio [228]. Similarly, a polyphenol-rich cranberry extract improved metabolic derangements induced by a high fat/high sucrose diet in mice, in concomitance with reduced intestinal inflammation and oxidative stress and a shift in gut microbiota with an increased abundance of the mucin-degrading *Akkermansia* spp. [231]. Interestingly, the transplantation of the fecal microbiota from resveratrol-treated mice, characterized by an enrichment in Bacteroidetes, to high fat diet-fed obese mice was capable of improving the intestinal barrier function, in association with decreased weight gain and inflammation and increased insulin sensitivity and lipid metabolism [232]. In a murine model of experimentally induced colitis, polyphenol-rich tea extracts from *Camellia sinensis* and *Litsea coreana* increased the abundance of potentially beneficial bacteria (e.g., *Faecalibaculum* and *Bifidobacterium*) and decreased the abundance of potentially harmful bacteria (e.g., *Bacteroids* and *Mucispirillum)*, restoring the decreased production of SCFAs [233]. These effects were accompanied by attenuated colon inflammation (through the inhibition of NF-κB) and oxidative stress and restored intestinal barrier function (reduced loss of TJ components, such as zonulin and occludin) [233]. In patients with inflammatory bowel disease, besides improving disease symptoms and reducing inflammation, a mango polyphenol extract modulated fecal microbial composition by increasing the abundance of *Lactobacillus* spp. and the production of fecal butyric acid [173].

Key bacterial enzymes, called polyphenol-associated enzymes (PAZymes, including tannase, quercetinase, gallate decarboxylase, esterase, and phenolic acid decarboxylase), are specifically involved in the potential and real prebiotic effects of polyphenols by promoting polyphenol metabolization [234] and have been involved in the bioactivity and health effects of polyphenols [235]. Indeed, through the action of these enzymes in the gastrointestinal tract, the poorly absorbed polyphenols can be utilized and transformed by beneficial gut microbes into bioavailable and bioactive phenolic metabolites that can act locally in the intestine and can be freely absorbed, thus reaching target organs and exerting health effects [229,236,237]. Therefore, the gut microbiota contributes to the bioavailability and bioactivity of unabsorbed polyphenols. Microbiota-derived SCFA may favor the intestinal absorption of polyphenol metabolites [238]. Some bacteria can utilize polyphenols to improve their fitness and their persistence in intestinal niches and can release phenolic metabolites that function as trophic factors used by other beneficial bacterial species, thus establishing syntrophic relationships capable of further shaping microbiota composition and function [234]. Accordingly, the growth-promoting activity of blueberry (poly)phenols on *Bifidobacteria* and *Lactobacilli* strains or of tannins on *Lactobacilli* species, such as *L. plantarum*, have been linked to the (poly)phenol-metabolizing abilities of these bacteria [239,240]. Interestingly, polyphenol polymers are often ingested as bound to cell wall matrix, thus reaching the colon in the form of fiber-associated polyphenols [241]. Studies have found that once the colonic microbiota ferment the fibers, SCFA are produced, and the “non-extractable” polyphenols are released and further metabolized. Therefore, local and systemic effects can be cooperatively provided by metabolites derived both from fiber and fiber-associated polyphenols. Fermentation of glycosylated polyphenols can also be a source of SCFA [242].

Although it is indisputable that the gut microbiota modulating activity of polyphenols may translate into beneficial intestinal outcomes including anti-inflammatory, antioxidant, immune, and barrier integrity protective effects, the specific role of polyphenol–gut microbiota crosstalk in NCGS remains unexplained and deserves further research. The capacity of polyphenols to re-establish the normal function of the mucosal epithelial barrier and its immunological response and to reduce oxidative stress may favor an increased abundance of gut bacteria with health benefits to the host, which underlies a polyphenol indirect prebiotic-like effect. Additional evidence is required to better understand the role of gut microbiota in the pathogenesis of gluten-related disorders and the clinical impact and therapeutic use of polyphenols in this setting.

## 6. Conclusions

This review has highlighted the potential multiple protective roles of dietary polyphenols, both in free and macromolecule-linked forms, in gluten-related diseases and in particular in NCGS.

Supposed molecular mechanisms of action of polyphenols in reducing the symptoms of NCGS are increasingly emerging. Polyphenols can exhibit a direct action of capturing gluten, the main trigger of gluten-related diseases such as NCGS, thereby reducing its bioavailability and associated toxic effects. Polyphenols can also dampen the toxic effects of gluten, both at the intestinal and systemic levels, by reducing oxidative stress and inflammation, by regulating the immune response, and by improving the health of the intestinal microbiota and the function of the intestinal barrier. Although much scientific evidence supports the protective role of polyphenols in gut health by preventing the onset of gluten-related diseases or reducing their symptoms, there is no evidence in humans with controlled studies to suggest their use in such pathologies. Future studies are needed before physicians and dieticians can recommend the use of dietary polyphenols to improve symptoms of NCGS as an alternative or as a GFD adjuvant.
